# Testing for an Effect of a Mindfulness Induction on Child Executive Functions

**DOI:** 10.1007/s12671-018-0923-2

**Published:** 2018-03-13

**Authors:** Anna Leyland, Lisa-Marie Emerson, Georgina Rowse

**Affiliations:** Department of Psychology, University of Sheffield, Cathedral Court, 1 Vicar Lane, Sheffield, S1 2LT UK

**Keywords:** Induction, Children, Executive function, Mindfulness, Experiment

## Abstract

Several sessions of mindfulness practice can exert positive gains for child executive functions (EF); however, the evidence for effects of a mindfulness induction, on EF for adults, is mixed and this effect has not been tested in children. The immediate effect of an age appropriate 3-min mindfulness induction on EF of children aged 4–7 years was tested. Participants (*N* = 156) were randomly assigned to a mindfulness induction or dot-to-dot activity comparison group before completing four measures of EF. A composite score for EF was calculated from summed *z* scores of the four EF measures. A difference at baseline in behavioural difficulties between the mindfulness induction and comparison group meant that data was analysed using a hierarchical regression. The mindfulness induction resulted in higher average performance for the composite EF score (*M =* 0.12) compared to the comparison group (*M* = − 0.05). Behavioural difficulties significantly predicted 5.3% of the variance in EF performance but participation in the mindfulness or comparison induction did not significantly affect EF. The non-significant effect of a mindfulness induction to exert immediate effects on EF fits within broader evidence reporting mixed effects when similar experimental designs have been used with adults. The findings are discussed with consideration of the extent to which methodological differences may account for these mixed effects and how mindfulness inductions fit within broader theoretical and empirical understanding of the effects of mindfulness on EF.

Executive functions (EF) are critical for child social and emotional competence as they allow modulation of emotions, thought and behaviour and are positively linked to academic attainment and social-emotional functioning in children (Kochanska et al. 2001). EF enable goal pursuit through sustaining and redirecting attention to goal-related tasks and inhibiting pre-potent (and often emotional) responses that may threaten goal attainment. Typically, EF are conceptualised as formed of three distinct but highly related cognitive processes: inhibitory control, cognitive flexibility and working memory (Miyake et al. 2000). Targeted interventions, including mindfulness training, can enhance EF in children (Diamond and Lee 2011) but little is known about the dose effect of mindfulness or which aspects of mindfulness training exert beneficial effects. Abbreviated mindfulness training and experimental mindfulness inductions can enhance EF in adults (e.g. Keng et al. 2013) but there are no empirical reports testing the effect of mindfulness inductions on child EF.

Mindfulness is commonly operationalized as having two components: the self-regulation of attention and the possession of a particular orientation to experience (of openness, non-judgement and curiosity; Bishop et al. 2004). Mindfulness encourages practitioners to attend to present-moment experience (thoughts, emotions, body sensations) and to refocus attention on the present moment when the mind engages with cognitive (e.g. thinking about the past or future) or meta-cognitive (e.g. appraising thoughts) processes. Typically, mindfulness-based interventions (MBI) are delivered as weekly group sessions (e.g. 2.5 h) over 8 weeks incorporating experiential practices (e.g. mindfulness of breathing, body scan), group discussion and home practice (Crane et al. 2017). There is some emerging but inconclusive empirical evidence for the effect of MBI on EF in children. Flook et al. (2010) demonstrated gains in parent and teacher reported EF for those children with low baseline EF following an 8-week MBI compared to a silent reading control. A longer 12-week MBI was compared to a social responsibility programme and found improved reaction times but not accuracy for two behavioural measures of EF (Schonert-Reichl et al. 2015). Another 12-week MBI was compared to a waitlist control group on three measures of EF and found no significant differences between groups (Flook et al. 2015). An 8-week MBI (90 min weekly plus home practice) delivered to children and their parents reported greater gains on attention regulation for children in the mindfulness group compared to a waitlist control (Felver et al. 2017). The evidence suggests mindfulness may exert an effect on EF in children particularly when reaction times are measured, when baseline EF is low and for attention regulation.

The executive control of attention (Shapiro et al. 2006) and decentring (the ability to observe rather than judge present-moment experience) are two proposed mechanisms through which mindfulness may enhance EF. The executive control of attention toward goal directed stimuli relate most strongly to the cognitive flexibility component of EF and so mindfulness may exert a benefit on EF via direct improvements to cognitive flexibility (Miyake et al. 2000). Comparatively, decentring is an emotion regulatory strategy (Gross 2002) and so mindfulness training may benefit EF by downregulating emotions that at high levels can impair EF (Zelazo and Lyons 2012). There is growing understanding of the effects of MBI on EF; however, less is known about the dose effect of mindfulness training and whether experimental inductions of mindfulness can produce effects.

The use of one-off experimental mindfulness inductions enables researchers to conduct fine-grained comparisons between mindfulness and viable control inductions, thereby extrapolating the specific effects of mindfulness (Tang et al. 2015). The effect of mindfulness inductions on EF in adults has been explored by researchers; however, the reported effects are mixed. Johnson et al. (2013) reported no significant differences on multiple measures of EF when a 25-min mindfulness of breathing induction was compared with sham meditation (explicit labelling of practice as meditation, guided breathing practice but no other mindfulness components) and a book-listening task. A similar study compared a 10-min mindfulness of breathing induction with an attention induction (e.g. participants imagine counting windows in their house) and an arithmetic exercise (Watier and Dubois 2016). The researchers found no difference in performance on EF (the emotional Stroop task) but for those with low trait mindfulness, the mindfulness induction increased executive attention compared to both comparison groups. There is also evidence of gains to EF following mindfulness inductions. Keng et al. (2013) demonstrated that a 10-min mindful acceptance induction reduced interference on the Stroop task compared to both a reappraisal induction and control group (no training). Similarly, a 5-min mindful eating task (compared to participants instructed to eat two raisins in 5 min) resulted in greater recovery in working memory performance following a stereotype threat task (Weger et al. 2012). The reason for the contradiction in the literature remains unclear. Some authors propose that to detect immediate changes following mindfulness inductions, measures of EF need to be sensitive to momentary lapses in attention (Johnson et al. 2013), or that the mindfulness induction is acting indirectly by altering state affect or fatigue (Zeidan et al. 2010) or is mediated by the induction of a mindful state (Mahmood et al. 2016).

Three published papers report the effects of a mindfulness induction with child participants. In one study, children aged 9–14 years participated in a surprise speech task and were given false negative feedback from peers to induce negative mood before being guided to ruminate on their experience (Hilt and Pollak 2012). They were then randomised to complete an 8-min guided practice of mindfulness, problem solving or distraction. The authors reported an equal effect of the mindfulness induction to the distraction activity in reducing rumination but both activities were significantly more effective than problem solving to reduce rumination. A second study reported the effects of a 10-min mindfulness induction compared to quiet play on children aged 7–9 years on measures of arousal, mood and social dominance (Nadler et al. 2017). The authors reported no significant effects of experimental condition on mood or social dominance but mindfulness resulted in reduced arousal (i.e. increased calmness) compared to those playing quietly, in two samples (Cohen’s *d* = 0.60; 2.40). A final study by Lim and Qu (2017) measured the effect of a 15-min mindfulness induction compared to an active control induction (e.g. dancing, singing, counting) on several measures of attention of 122 children aged 4–6 years. No between-group differences were found for orienting, alerting or executive control of attention but changes were reported in the reduction of attention scope bias (i.e. a preference for global or local processing at pre-induction) for those in the mindfulness induction group. This finding gives some impetus for further investigation of mindfulness inductions using experimental designs with children. There are however gaps in current understanding including whether the methodology is feasible in a younger child sample and if mindfulness inductions can exert an effect on EF in children.

The central goal of this study was to assess the immediate effects of a mindfulness induction on EF compared to a comparison activity. We hypothesise that children participating in the experimental mindfulness induction will subsequently achieve higher EF scores than those participating in the comparison (dot-to-dot activity) group.

## Method

### Participants

The study used a between-group design with two conditions: mindfulness induction and a comparison group. Scores on post-intervention tasks that measure EF formed the dependent variable. Children aged 4–7 years with English as a first language were recruited from three schools in a city in the North of the UK (*N* = 159). Informed consent was obtained from all parents and assent from children participating in the study. The study had 93% power to detect a medium effect size (*d =* 0.5). Children were assigned to conditions using block randomisation aiming for equal condition allocation between schools. Two children did not assent to participate in the study and one child could not understand the task instructions (*N =* 156). Both groups had a mean age of 6 years old (SD = 11 months) with the mindfulness group (*n =* 80) having 45% female participants and the comparison group (*n =* 76) 46% female. Both conditions had 11% of participants in receipt of free school meals (used as an indicator for social deprivation).

### Procedure

Data collection occurred individually in each school in a quiet room. Children were told they were going to ‘play some games’ and asked to give their assent to participate. Participants were randomly assigned to one of three activities and either watched a cartoon on a laptop computer or coloured in a line drawing. Both the experimenter and the participant then followed instructions for the mindfulness induction, played through laptop computer speakers or completed a dot-to-dot drawing task. For the mindfulness induction, the participant and experimenter were positioned on the ground facing each other around 1 m apart with a marble placed on the floor between them. Those children completing the dot-to-dot task were positioned at a table with the experimenter sitting adjacent to them. After completion of the induction, the experimenter followed a standardised procedure and script to administer the four tasks measuring EF, in the following order: Tower of Hanoi, head, toes, knees and shoulders (HTKS), delay of gratification and backward digit span. Data collection lasted around 35 min.

#### Depletion Activity

Prior to engaging in the induction, children were randomly assigned to participate in one of three depletion activities: watching a fast or slow pace cartoon, or colouring. The activities aimed to place differing demands on cognitive resources and had previously been reported to deplete EF in children aged 4 years, specifically with those watching the fast-paced cartoon having poorer performance on EF relative to those colouring (Lillard and Peterson 2011). The cartoons were presented on a laptop computer and each depletion activity lasted for 9 min.

#### Inductions

The mindfulness induction was a brief version (3 min and 20 s) of the ‘sound in space game’ (Greenland 2010, p. 95) pre-recorded by a clinical psychologist experienced in teaching mindfulness to children. The game uses several foci of attention: visual (marble), auditory (chime) and sensory (movement of breath). The induction is age appropriate and used within an established mindfulness programme for children (Flook et al. 2010). The comparison group completed a number of dot-to-dot drawings for the same duration. The comparison group activity was designed to match the general aspects of the experimental mindfulness induction (e.g. experimenter-participant interactions) so that mindfulness-specific aspects of the induction, e.g. monitoring of sensory experience, could be empirically tested.

### Measures

The Strength and Difficulties Questionnaire-parent (SDQ-parent) is a 25-item behavioural screening tool assessing the behaviours, emotions and relationships of children age 4–16 years (Goodman 1997). Items are scored on a three-point scale (not true, somewhat true and certainly true) with parents rating their child’s behaviour over the last 6 months. The measure is formed into four subscales of behaviour difficulties (peer relationship problems, hyperactivity/inattention, conduct problems, emotional symptoms) and the pro-social behaviour subscale. The SDQ-parent has shown a good reliability in a large community sample for all subscales including pro-social behaviour (*α* = 0.65) and behavioural difficulties (*α* = 0.82; Goodman 2001). The present study included the score for behaviour difficulties and pro-social behaviour as indicators of baseline functioning in key areas associated with EF: attention, social functioning and behavioural self-regulation.

The Tower of Hanoi (Simon 1975) was used to assess planning and working memory (Welsh et al. 1999). Three wooden pegs on a base were described as trees and the two discs were positioned on the left peg. The discs were described as ‘Mummy’ (small red disc) and ‘Daddy’ (large blue disc) monkeys who wanted to move to their sleeping tree (the right peg; Welsh et al. 1999). A visual aid depicting the final configuration of monkeys and trees was also presented on an A4 paper. Children were told three rules to follow: one monkey could be moved at a time; the Daddy monkey could never go on top of the Mummy monkey and the monkeys always needed to be on a tree. A score of one was given if all rules were followed to achieve the final disc configuration and zero if rules were broken or the task not completed. The task is valid with young children and has good test-retest reliability (*α* = 0.72; Gnys and Willis 1991).

The HTKS primarily assesses inhibitory control and behaviour regulation but also measures working memory and attention (Ponitz et al. 2009). This task consists of three progressive rounds of auditory commands where the child is required to inhibit a dominant motor response (e.g. ‘When I say touch your head, I want you to touch your toes, and when I say touch your toes, I want you to touch your head’). Following a brief practice, children progressed to the first round. Responses were scored: (0) incorrect, (1) initially incorrect but then corrected or (2) correct. If children scored 10 points on their first round, they progressed to round two, where two further instructions (shoulders-knees) were added; those who scored 14 or more in round two progressed to round three, where the rule was changed (head-shoulders, knees-toes). HTKS has good reported validity with parent and teacher reports of child EF (Ponitz et al. 2009).

The delay of gratification task is a sustained delay task where children are given the option of immediately eating a small portion of a chosen snack (sweet crackers, jelly beans, chocolate buttons) or waiting an unknown length of time to eat a larger portion (Mischel et al. 1989). Ten pieces of the snack were placed on one plate and two pieces on the other, with a hand bell between the two plates arranged on a table in front of the seated child. Children were told that the experimenter was about to leave the room and that they could ring the bell at any time to signal the experimenter to return, at which time they could eat the smaller plate of snacks. Alternatively, they could choose to wait an unspecified length of time for the experimenter to return when they would be able to have the larger plate of snacks. The experimenter recorded the time between leaving the room and the bell ringing or when the child ate the snack, or they returned to the child after 330 s. Delay of gratification tasks, including the sustained delay paradigm used here, have good convergent validity (*r* = .21, 95% CI = 0.09, 0.32; Duckworth and Kern 2011).

The backward digit span task (Wechsler 1949) measures the phonological loop and central executive components of working memory (Morra 1994). The task requires children to repeat back in reverse order a verbally presented series of digits. Fifteen number strings of two to six digits were created for use in this experiment and were scored according to established guidance (Carlson 2005) with the highest level of success recorded between 0 and 5. The task finished at a score of 5 or after three consecutive incorrect responses. The measure shows good convergent validity and good test-retest reliability (*α* = 0.62; Gathercole and Pickering 2000; Gathercole et al. 2004)

### Data Analyses

To assess differences on pre-induction variables, independent *t* tests and chi-square analysis were used. Any baseline differences can be assumed to have occurred by chance as the participants were randomly assigned to induction groups, but where the baseline measure interacts differently with, or has a theoretically or empirically grounded association with, the main outcome; the appropriate analysis is the multiple regression (Miller and Chapman 2001). A composite score for EF was calculated by summing transformed *z* scores for each of the four measures of EF (Cronbach’s *α* = 0.66). A hierarchical regression was conducted to determine the effect of the induction on the composite EF score and the relative effect of any predictors. Variables were entered as predictors because of their potential direct effect on the outcome or because they may have been interacting with the induction to effect the outcome.

## Results

### Baseline Comparisons

Participant demographics and measures taken pre-induction are presented in Table 1. Baseline differences between induction groups were assessed and groups were equal for the following: age *t*(154) = 0.10, *p* = .467, gender *x*^2^(1) = 0.04, *p* = .873 and free school meals *x*^2^(1) < .01, *p* = 1.000. The comparison group had a significantly higher score than the mindfulness group for behavioural difficulties (*t*(149) = 3.00, *p* = .003) and lower score for pro-social behaviour (*t*(149) = − 3.57, *p* < .001). On further examination, behavioural difficulties were interacting differently with performance on measures of EF, with a moderate significant negative correlation for the comparison group (*r =* − .48, *p <* .001), and a positive but non-significant association for the mindfulness induction (*r =* .04, *p =* .763). There was no significant effect of the depletion activity across the participant group for its effect on the composite score of EF (*F*(149.2) = 1.24, *p* = .292). Consequently, the effect of the depletion activity was excluded from further analysis.

**Table 1:** Participant demographics, child behaviour and mean executive function scores for mindfulness induction and dot-to-dot comparison group. Standard deviations are shown in parentheses

	Mindfulness *n =* 80	Comparison group *n =* 76
Age months	72.41 (10.7)	72.58 (10.5)
Female	45%	46%
Free school meals	11%	11%
SDQ pro-social behaviour	6.69 (3.75)	4.53 (3.68)
SDQ behavioural difficulties	8.77 (7.20)	12.18 (6.80)
Delay of gratification time waited seconds	263.77 (121.00)	244.33 (132.72)
Backwards digit span	1.79 (0.87)	1.81 (1.09)
Head toes knees and shoulders	45.18 (15.58)	42.77 (18.37)
Tower of Hanoi completed successfully	55%	55%

### Effects of Mindfulness Induction on EF

Results of measures of EF for the mindfulness and comparison groups are reported in Table 1. The mindfulness group score for EF was higher (*M =* 0.12 SD *=* 2.71) than the comparison group (*M =* − 0.05 SD *=* 2.97). The multiple regression analysis (step-one predictor of behavioural difficulties; and step-two predictor of induction group) significantly predicted EF and could explain 5.3% of variance in the outcome (*F*(2,144) = 4.05, *p = .*019; Table 2). Step-one predictors significantly predicted EF (*F*(1,145) = 7.85, *p* = .006), but the addition of the induction group as a predictor was not significant and did not increase the predictive value of the model. An experimental mindfulness induction had no significant impact on performance on measures of EF when compared to a dot-to-dot comparison group activity.

**Table 2:** Hierarchical Multiple Regression analysis predicting EF from induction and behavioural difficulties

Variable	Composite EF score			
	Step 1		Step 2	
	*β*	95% CI	*β*	95% CI
Constant	0.96	0.15, 1.77	1.13	0.10, 2.16]
Behavioural difficulties	− 0.23*	− 0.15, − 0.03	− 0.24*	− 0.16, − 0.03
Induction			− 0.04	− 1.19, 0.69
*R* ^2^	0.05		0.05	
*F*	7.85*		4.05*	
Δ*R*^2^			0.00	
Δ*F*			0.28	

*p < .05

## Discussion

The current study tested whether a 3-min mindfulness induction could exert an immediate effect on EF in children aged 4–7 years. Whilst those in the mindfulness group performed better on a composite measure of EF than those in the comparison (dot-to-dot activity) group, it is notable that this outcome was not due to participation in either the mindfulness induction or dot-to-dot comparison activity but rather was a result of the groups having significantly different scores for behaviour difficulties, which were interacting differently with the main outcome. This outcome does not support our hypothesis that a mindfulness induction would exert an immediate benefit on EF; however, the null findings are in line with current conflicting evidence for an immediate effect of a mindfulness induction on EF in adults.

The current evidence for an effect of a mindfulness induction on EF includes reported non-significant (e.g. Johnson et al. 2013) and significant positive effects (e.g. Keng et al. 2013). Similarly, there are differential effects reported when mindfulness inductions have been tested with children on other outcomes. Mindfulness was equally effective to distraction but superior to problem solving in lowering state rumination (Hilt and Pollak 2012). Arousal was significantly lower following a mindfulness induction when compared to quiet play but there was no effect of induction on mood or social dominance (Nadler et al. 2017). Similarly, although mindfulness effected change in biases in attentional scope, no effect was reported on orienting, alerting or executive attention (Lim and Qu 2017). Supporting this, evidence with adults supports the effectiveness of distraction as an approach for emotion regulation, which may be equitable to the regulating effect of mindfulness (Broderick 2005). The current study used a dot-to-dot task as the comparison activity that may have acted in a similar way to other distraction activities and could have masked any effects of the mindfulness induction on EF. Therefore, the results of the current study fit within the differential effects reported in this field and may be a result of the selected comparison activity eliciting an equitable effect to the experimental induction on child EF.

Mindfulness inductions can be used empirically with high levels of control to compare the effects of a mindfulness practice with comparison activities (Keng et al. 2011). The contribution of this experimental approach is therefore valuable to the literature, particularly at a time when researchers are aiming to better understand under what instances and through what mechanisms mindfulness may effect change (e.g. Gu et al. 2015). However, the deviation of mindfulness as an induction from the more traditional forms of training in mindfulness, such as MBI, is notable. Specifically, most mindfulness inductions are an isolated extracted component of a much broader MBI package, with the aim of the former to elicit immediate and short-term change and the latter a longer lasting change. The present findings did not detect an effect of the mindfulness induction on EF, which is in line with the differential findings of existing evidence. The reason mindfulness inductions do not consistently elicit immediate effects on EF may be due to methodological differences between experimental designs. Anecdotally, a review of the literature demonstrates differences in methodologies based on the mindfulness induction (e.g. duration, instructions), measurement of EF (e.g. the Flanker task, the Stroop test) and comparison activity (e.g. distraction, no instruction). The present study selected a mindfulness induction practice that primarily targeted attention regulation, one of the core components of mindfulness, which is highly pertinent when the target outcome is EF (Bishop et al. 2004). Additionally, several measures of EF were taken so as to cover multiple components of EF and the comparison activity, a dot-to-dot drawing task, required focussed attention and problem solving. These aspects of the method could have impacted on the absence of any detected effect of the induction; however, without a comprehensive review of the evidence, it is difficult to determine the extent to which methodological differences may impact on the detection and strength of effects.

An alternative explanation of the findings may be that mindfulness inductions are not always sufficient to effect change in EF, particularly for children. This explanation is particularly plausible in the context of what is known about the effects of MBI on EF for children. Specifically, where significant effects have been reported, these are often caveated by other factors, such as that only those with low baseline EF improved significantly post-intervention (Flook et al. 2010). This interpretation of the findings brings forth a bigger question across the broader literature surrounding mindfulness; under what conditions does mindfulness elicit demonstrable effects? MBI offer a broader and more comprehensive mindfulness training with more time for experiential practices and mindfulness-based education. There is growing empirical evidence of which components of MBI significantly contribute to effecting change. For example, the extent to which participants of MBI practice mindfulness at home positively impacts on changes post-intervention (Parsons et al. 2017). There is little evidence exploring why mindfulness inductions may not deliver a hypothesised change, although many authors anecdotally comment on methodological limitations such as the choice of outcome measure (Johnson et al. 2013) or induction practice (Ridderinkhof et al. 2017). Beyond these interpretations, one may speculate that mindfulness inductions differ from MBI as participants are expected to complete an experiential mindfulness practice without any context, such as supporting education or discussion, and without any internally driven motivation to engage with mindfulness. Three key components of mindfulness practice are proposed as follows: intention, attitude and attention (Bishop et al. 2004). Both the attitude with which someone approaches a mindfulness practice, particularly one that is open and non-judgemental, and the regulation of attention toward present-moment experience can be directly targeted by the instructions of the mindfulness induction. It is more difficult to direct an individual’s intention, which determines ones motivation to practice, such as a desire to alleviate difficult emotional or physical experiences. Participants of mindfulness inductions are often only motivated to participate in an experiment and are ordinarily unaware of the experimental aims, including the presence of a mindfulness practice, until the end of the experimental session. The motivation to practice mindfulness may be being indirectly measured through reports of amount of home practice in MBI, demonstrating a link between motivation to practice, actual practice and outcomes (Parsons et al. 2017).

The age of the sample in the current study may have introduced some additional barriers to the effectiveness of the mindfulness induction, in particular because children were at a pre-operational stage of development. Constructs highly relevant to mindfulness, such as decentring, may necessitate sufficient existing capacities of meta-cognition, attention, introspective awareness, memory and verbal fluency (Black 2015), which are unlikely to have fully developed in children under 8 years old (e.g. Flavell et al. 2000). Although these capacities can be enhanced in children by MBI (e.g. Felver et al. 2017; Flook et al. 2010), there is no empirical measure of the effect of a mindfulness induction on these constructs in children and therefore, it is difficult to elaborate on the effect that the developmental stage may have had on the null findings of this study.

The performance on the measures of EF was in part explained by parental reports of child behaviour difficulties. There is substantial evidence demonstrating deficits in EF as predictors of low pro-social behaviour and increased behavioural difficulties (e.g. Hughes et al. 2000). The causative nature of EF on behaviour is unsurprising, given that components of EF such as inhibitory control will directly determine one’s capacity to override pre-potent responses (e.g. to hit a peer) that do not fit with overriding goals (e.g. not to be violent). Baseline differences were identified between those allocated to the mindfulness induction and comparison activity. Specifically, in the comparison group, there was significantly higher behaviour difficulties overall and behaviour difficulties were negatively associated with EF. There are known associations between higher scores on the SDQ and other pertinent measures of child functioning including the following: speech and language difficulties (Beitchman et al. 1996) and other psychopathological conditions such as anxiety and depression (Muris et al. 2003). These factors were not measured in the present study and so the presence of between-group differences and the subsequent effect on EF is unknown.

## Limitations

The current study did not measure baseline or pre-induction EF, limiting interpretation of the findings with regard to within-participant change following induction and the extent to which causal inferences can be made. The measurement of pre-induction EF present some challenges; in cases where novelty of task is important (e.g. delay of gratification), learning effects are high (e.g. Tower of Hanoi success or fail) and automatization can occur (e.g. HTKS), and where further extension of test duration is undesirable (e.g. for a child participant group (see Beck et al. 2011; Müller et al. 2012)). Addition of baseline testing sometime prior to the experimental testing session may reduce or negate some of these effects, but may not be practically feasible (e.g. time constraints) and normal development over time could mask the effects of the induction if baseline and experimental measures are taken too far apart. Taking a different measure of EF at pre-induction (e.g. Flanker Task) as an indicator of pre-induction performance may also overcome this, with evidence demonstrating that one measure of EF can predict performance on other EF measures (Senn et al. 2004). This approach is limited as association between EF components are complex, not always highly correlated and the associations between EF components change across development (Best et al. 2009). A particular strength of this experiment was a large sample size and the use of block randomisation to induction group. Disappointingly, there were baseline differences identified in parental reports of child behaviour; however, these were addressed statistically and the findings were interpreted with consideration of these differences. Additionally, it is acknowledged that unequal allocation of baseline characteristics is common in small and moderate sample sizes (Shadish et al. 2002) and the use of randomisation to induction leads us to conclude that these between-group differences occurred by chance (Miller and Chapman 2001).

The experiment benefitted from the use of multiple behavioural measures of EF, identified as a limitation of existing research (e.g. Black 2015); however, the use of multiple measures extended the testing period and therefore prescribed that the effects of the short mindfulness induction (3 min) needed to extend over a relatively long testing time (approx. 25 min), if effects were to be detected. Additionally, each EF task presented participants with some cognitive, and for some tasks, affective load (e.g. delay of gratification) potentially masking the effect of the mindfulness induction over time. Further research could prioritise one aspect of EF requiring a single measure to reduce the impact of the EF tasks on each other and isolate the effect of the mindfulness induction on the outcome.
